# Identification, Superantigen Toxin Gene Profile and Antimicrobial Resistance of Staphylococci Isolated from Polish Primitive Sheep Breeds

**DOI:** 10.3390/ani12162139

**Published:** 2022-08-20

**Authors:** Jolanta Karakulska, Marta Woroszyło, Małgorzata Szewczuk, Karol Fijałkowski

**Affiliations:** 1 Department of Microbiology and Biotechnology, Faculty of Biotechnology and Animal Husbandry, West Pomeranian University of Technology in Szczecin, Piastów 45, 70-311 Szczecin, Poland; 2 Department of Ruminant Science, Faculty of Biotechnology and Animal Husbandry, West Pomeranian University of Technology in Szczecin, Janickiego 29, 71-270 Szczecin, Poland

**Keywords:** staphylococci, *gap* gene, primitive sheep breed, antimicrobial resistance, enterotoxin, exfoliative toxin, toxic shock syndrome toxin 1, *Alu*I enzyme, *Hpy*CH4V enzyme

## Abstract

**Simple Summary:**

Current knowledge regarding the occurrence, differentiation, and pathogenicity of staphylococci isolated from animals is mainly based on clinical isolates, but little is known about staphylococci colonizing healthy animals. Even less is known about staphylococci that colonize sheep. While considering the microbiota inhabiting the primitive native breeds of sheep from the ecological/agritourism farm, there is no such information at all. The current study aimed to identify staphylococcal species isolated from healthy sheep of two native breeds (Świniarka and Wrzosówka) kept on the same ecological/agritourism farm. Furthermore, the prevalence of selected toxin genes and antimicrobial resistance was also determined. A total of 127 coagulase-negative staphylococci were identified from 61 sheep (33 of Świniarka) and (28 of Wrzosówka). From 1 to 4 staphylococcal isolates were obtained from one sheep. *Staphylococcus aureus* was not identified in any of the samples. In total, seven different species were identified. Over 60% of staphylococci were resistant to at least one antimicrobial of therapeutic importance and over 77% possessed from 1 to 7 genes encoding different toxins. The findings of this study prove that toxigenic and antimicrobial-resistant coagulase-negative staphylococci can colonize the nasal cavity of healthy sheep of primitive, native breeds.

**Abstract:**

The study aimed to analyze staphylococcal microbiota of the nasal cavity of the primitive sheep breeds Polish Świniarka and Wrzosówka kept on the same ecological farm. The research included the identification of staphylococcal species, evaluation of the prevalence of genes encoding enterotoxins, staphylococcal enterotoxin-like proteins, exfoliative toxins, toxic shock syndrome toxin 1, and detection of antimicrobial resistance. From 61 swab samples gathered from Świniarka (33) and Wrzosówka (28) healthy sheep, 127 coagulase-negative staphylococci (CoNS) were isolated. Based on PCR-RFLP analysis of the *gap* gene using *Alu*I and *Hpy*CH4V enzymes, the isolates were identified as: *Staphylococcus xylosus* (33.9%), *S. equorum* (29.1%), *S. arlettae* (15%), *S. warneri* (9.4%), *S. lentus* (7.9%), *S. succinus* (3.9%) and *S. sciuri* (0.8%). Three of these species, *S. lentus*, *S. succinus,* and *S. sciuri*, were detected only from the Świniarka breed. It was found that 77.2% of isolates harbored from 1 to 7 out of 21 analyzed genes for superantigenic toxins. The greatest diversity of toxin genes was recorded for *S. equorum* (16 different genes). The most prevalent gene was *ser* (40.2%). The incidence and number of resistances to antimicrobials were found to be bacterial species but not sheep breed dependent. The highest percentage of resistance was found for *S. sciuri*. The most frequent resistance was observed to clindamycin (45.7%). The findings of this study prove that toxigenic and antimicrobial resistant CoNS can colonize the nasal cavity of healthy sheep.

## 1. Introduction

The primitive, native breeds of animals are of great importance due to the role they play in the development of regions where they are kept [1,2,3,4]. As an example, the indigenous breeds of small ruminants fulfill a natural landscape, ethnographic and socio-cultural function, which is considered to be a testimony to the traditions and culture of local communities [5,6,7]. Interest in native herbivorous animal breeds has been growing in recent years because such a type of breeding can contribute to the management of vegetation and the care of landscapes [2,8]. In contrast, industrial methods of animal husbandry, including sheep herding, have led to global production being based on a small number of high-performing breeds. Meanwhile, the biodiversity of farm animals is an essential component of the sustainable development of agricultural production and rural areas [9,10].

Poland has a long tradition in the fields related to the protection of genetic resources of farm animals, most of which have been included in the List of Global Genetic Resources [11]. Currently, 18 old native breeds of sheep are covered by the Program for the Protection of Genetic Resources of Farm Animals [12], including Wrzosówka and Świniarka [13]. 

Wrzosówka and Świniarka breeds are characterized by extremely high disease resistance, longevity, easy adaptation, resistance to difficult environmental and climatic conditions as well as low feeding and breeding requirements [14,15]. These features make them useful for the maintenance of local landscapes, the preservation of the natural environment, and care of nature-protected areas [14,16,17]. Sheep of these breeds are short in height and characterized by a marked sexual dimorphism and mixed two-fraction wool, which is grey in Wrzosówka and white in Świniarka. Both breeds are considered as of relatively low slaughter value, although their meat is characterized by high-quality and unique taste [12,18].

Wrzosówka is one of the oldest breeds of domestic sheep in Poland. Sheep of this breed have very well-developed maternal abilities and their reproductive cycle is characterized by aseasonality (possibility of three lambings per year), high fertility of 175–180%, and early breeding maturation. Their excellent quality wool is mainly used to produce velor [18,19,20]. Due to the deepening decline in the population of the Wrzosówka sheep and the threat of their extinction, the National Research Institute of Animal Production in Poland decided to restore this breed in the 1970s on the base of a total of 160 ewes and 27 rams [12,18]. Currently, in Poland, there are 105 flocks with a total of 9584 animals [11]. 

The Świniarka breed is characterized by breeding seasonality, average fertility of 120% in a single litter, and low fertility of about 75% in difficult environmental conditions. Sheep of this breed have loose, sparse, and mixed wool [21]. Due to the low population of Świniarka sheep at the end of 1999 (180 ewes and rams), this breed was classified in Poland as threatened by extinction. In consequence, the original genotype of the Świniarka breed has been enlarged and restored on the base of 17 ewes and 3 rams. At present, in Poland, there are 36 flocks with a total of 2805 animals [11]. 

Local breeds, less frequently used in intensive systems but still preserved in local territories, represent an important resource for animal biodiversity [22]. As suggested by Papachristoforou et al. [23], the available strategies to increase the value of local breeds with special attention to sheep and goats can be grouped into three interconnected categories: (i) linking local breeds to traditional products and/or to tourism/agritourism activity, (ii) promoting the use of local breeds in specific farming systems (e.g., organic production, low-input, and hobby farms or through the preservation of grazing-silvo-pastoral systems) and (iii) implementing general strategies (e.g., marketing, legislation, organization of stakeholders and raising public awareness).

According to the 16S rRNA analysis, the *Staphylococcus* genus is divided into 62 species and 30 subspecies [24]. Most of the staphylococci constitute a significant part of the microbiota and most of them are harmless and reside normally on the skin and mucous membranes of nostrils and respiratory system of humans and animals [25,26,27]. The pathogenic capacity of staphylococci is attributed to a combination of their invasive properties, the production of virulence factors, including extracellular toxins, and drug resistance. Staphylococcal toxins include classical enterotoxins (SEs; SEA to SEE, SEG to SEI, SER), enterotoxin-like (SEl) proteins, exfoliative toxins (ETA to ETD), toxic shock syndrome toxin 1 (TSST-1), hemolysins and Panton-Valentine leukocidin (PVL) [27,28]. Moreover, different species of staphylococci have been suggested as a reservoir of antimicrobial resistance which can be transferred to other strains, including *Staphylococcus aureus*, making it resistant to multiple agents [26].

The current knowledge regarding the occurrence, differentiation, and pathogenicity of staphylococci isolated from animals is mainly based on clinical isolates, but still little is known about staphylococci colonizing healthy animals, including their nasal cavity [29,30]. Even less is known about staphylococci that colonize sheep. While for microbiota inhabiting the primitive native breeds of sheep, there is no such information at all. A similar lack of information concerns prevalence of antimicrobial resistant strains among coagulase-negative staphylococci (CoNS) colonizing sheep. Even if such studies are conducted, most of them concern staphylococci isolated from mastitis [31]. However, CoNS colonize skin, noses, urogenital and intestinal tracts of animals as their natural microbiota and could be reservoir of various antibiotic resistance genes, including *mec*A gene, which encode resistance to β-lactam antibiotics. The *mec*A gene has been detected in number of CoNS from various animal species, including sheep [32,33]. Furthermore, the CoNS strains, according to Taponen and Pyörälä [34] could be even more resistant to antimicrobials in relation to *S. aureus* and may present a characteristic of multidrug resistance.

Increasingly, ecological and agritourism farms keep sheep covered by conservative breeding due to previously mentioned features like good health, easy adaptation, resistance to harsh environmental conditions, longevity, fertility, maternal traits [10,35], and low feed requirements [35]. In addition, great attention is attached to the highest quality products obtained from animals, such as wool, meat, milk, and hides [14,36,37].

It is worth emphasizing that animals kept on such farms have often direct and close contact with people, including young children and elders. Therefore, the topic discussed in this work, which considers the potential pathogenicity of staphylococci isolated from healthy sheep from agritourism farm seems to be very important because of the possibility of transfer of microorganisms between humans and animals. Because of the high transmission potential of staphylococci, it was also assumed that the microbiota of the nasal tract of sheep which were kept on the same farm and under the same conditions will not be different between breeds. Furthermore, taking into account that the animals were kept on the ecological farm and were clinically healthy, well-fed, and never treated with any antimicrobials, it was also assumed that the isolated bacteria will be characterized by a low degree of antibiotic resistance and a low pathogenicity potential, understood as the ability to produce superantigenic exotoxins. 

The current study aimed to identify staphylococcal species isolated from healthy sheep of two native breeds kept on the same ecological and agritourism farm. Furthermore, the prevalence of selected superantigen (SAg) genes (encoding staphylococcal enterotoxins, staphylococcal enterotoxin-like proteins, exfoliative toxins, and toxic shock syndrome toxin 1) and antimicrobial resistance was also determined among collected staphylococcal isolates. This knowledge seems to be essential to provide background information on the incidence, species diversity, and potential pathogenicity of commensal staphylococcal biota.

## 2. Materials and Methods

### 2.1. Sheep and Their Maintenance Conditions

**Figure 1:**
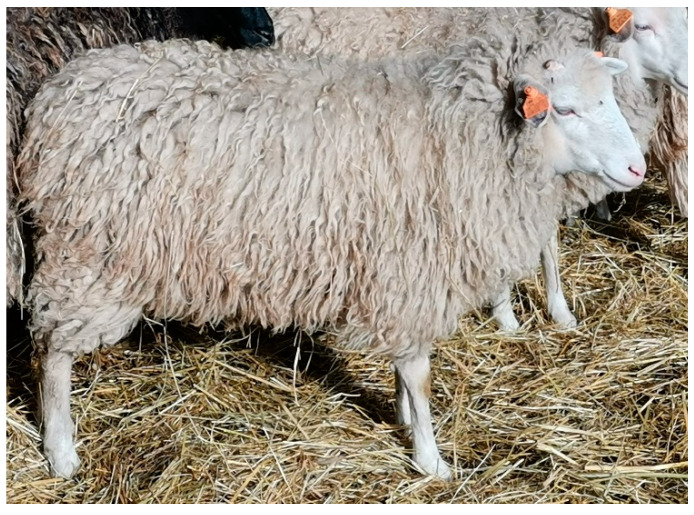
Świniarka sheep.

**Figure 2:**
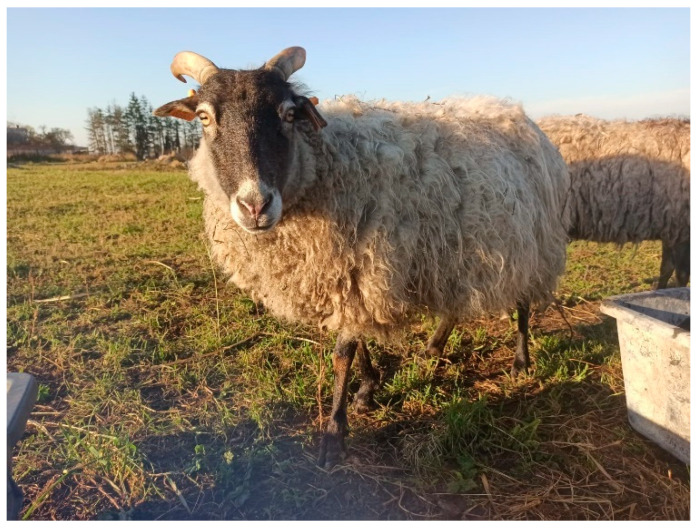
Wrzosówka sheep.

The sheep included in the study belonged to one herd of 61 female individuals aged from 6 months to 12 years, including 33 Świniarka (Figure 1) and 28 Wrzosówka (Figure 2) breed. Sheep were housed under the same conditions on a certified organic farm (controlled by the Agro Bio Test Ltd., Warsaw, Poland) ensuring animal welfare standards and running an agritourism activity. This farm is located at the West Pomeranian Voivodeship in the Lower Oder Valley Landscape Park. This area consists of the largest fluorogenic fens in Western and Central Europe with unique flora and fauna compared to the valleys of other European regions. Due to its natural, historical, and cultural values, the area is protected as a part of the European Ecological Network “Natura 2000”. To prevent the depletion of the valuable nature of the Landscape Park (e.g., xerothermic grasslands), grazing of large and small ruminants, including Wrzosówka and Świniarka sheep, has been conducted for several years.

From April to November sheep were grazed on pasture (Figure 2) all day, whereas during autumn and winter the animals were kept in buildings with partly wooden and partly brick structures, where they resided in a group of pens measuring 8 m × 9 m. The animals had dry lairs covered with straw (Figure 1) and access to water and hay at will. Additionally, they received oatmeal during autumn-winter period. Sheep also have access to licks all year round.

### 2.2. Sampling 

Swabs cultures were obtained from the nasal cavities (from both nostrils) of 61 healthy sheep (33 of Świniarka and 28 of Wrzosówka) in March 2021. The sterile swabs were introduced separately into each nostril to a depth of 3 cm and rolled on the mucosal membranes for 5 s after carefully cleaning the front of the nostrils and nasal mucous membrane with a disinfectant (the swabs used in our work were collected by a veterinarian taking care of an organic herd as part of routine veterinary care of the sheep). Next, the swab samples were transported to the laboratory in Stuart’s medium (Oxoid, Basingstoke, UK) at 4 °C and inoculated on selective agar within 24 h as described below. 

### 2.3. Isolation and Phenotypic Identification of Staphylococci

Swabs were streaked onto the Mannitol Salt Agar medium (MSA, BioMaxima, Lublin, Poland) and incubated at 37 °C for 48 h. Each morphologically different colony of bacteria was taken for further analysis. Phenotypic characteristics of isolates were assessed based on bacterial morphology in Gram-stained microscopic preparations, oxidase, and catalase production, the ability to decompose mannitol on Mannitol Salt Agar (BioMaxima, Poland), and hemolytic activity on Columbia Agar Base (BioMaxima, Poland) with 5% addition of defibrinated sheep blood (Graso, Starogard Gdański, Poland). The presence of the clumping factor (CF) and coagulase production was determined by the slide and tube method, respectively, using rabbit plasma (BioMaxima, Poland), according to the manufacturer’s instructions. The species identification of isolates was performed by the methods described below. 

### 2.4. DNA Extraction 

Bacteria were plated onto Columbia Agar Base with 5% sheep blood (Graso, Poland) and cultivated for 24 h at 37 °C. After incubation, one colony of each isolate was transferred into Trypticasein Soy Broth (BioMaxima, Poland) and incubated for 24 h at 37 °C. Then, the optical density (at 600 nm) of bacterial cultures was adjusted to 1.0. The total DNA was extracted from bacteria using the *GeneMATRIX Bacterial & Yeast Genomic* DNA Purification Kit (EURx, Gdańsk, Poland), according to the manufacturer’s instructions. 

### 2.5. Detection of gap Gene, mecA Gene and Superantigen (SAg) Genes

The *gap* gene and the *mec*A gene were detected using primers previously described by Yugueros et al. [38] and Murakami et al. [39], respectively. The presence of SAg genes encoding SEs, SEl, ETA, ETD, and TSST-1 toxins was determined by multiplex PCR with 5 different sets of primers as described by Zhang et al. [40], Jarraud et al. [41], Holtfreter et al. [42] and Fijałkowski et al. [43,44]. 

The PCR and each of five multiplex PCR reaction mixtures (12.5 µL) consisted of: 6.25 µL of GoTaq^®^ G2 Green Master Mix (Promega, Madison, USA), 0.5 µM (*gap* gene) or 0.25 µM (*mec*A gene) or 0.15 to 0.4 μM (SAg genes) of each primer (oligo.pl, Poland) and 1 µL of template DNA (20–50 ng). 

The PCR conditions were as follows: an initial denaturation of DNA at 94 °C for 2 min; 35 cycles (*gap* gene, SAg genes) or 30 cycles (*mec*A gene): denaturation at 94 °C for 20 s, annealing of primers at 50 °C for 45 s (*gap* gene) or 53 °C for 30 s (*mec*A gene) or 55 °C for 45 s, extension at 72 °C for 40 s; final extension at 72 °C for 5 min. PCR was performed in a peqSTAR thermocycler (Peqlab Biotechnologie GmbH, Erlangen, Germany). 

The DNA of the following control strains of *Staphylococcus aureus* was used: ATCC 43300 (*gap*, *mec*A) and ATCC 25923 (*gap*, no *mec*A) for determining *Staphylococcus* genus and methicillin resistance, A920210 (*eta*) [45], Col (*seb*, *selk*, *selq*) [45], FRI1151m (*sed*, *selj*, *ser*) [42], FRI137 (*sec*, *seh*, *sell*, *selu*) [45], FRI913 (*sea*, *sec*, *see*, *selk*, *sell*, *selq*, *tst-1*) [45], N315 (*sec*, *seg*, *sei*, *sell*, *selm*, *seln*, *selo*, *selp*, *tst-1*) [46], TY114 (*etd*) [45] and 8325-4 (no SAgs genes) [42] for SAg genes detection.

### 2.6. Staphylococcus Species Identification-PCR-RFLP of gap Gene

The *gap* gene amplification products were digested with *Alu*I (Fermentas, Waltham, MA, USA) and *HpyCH*4V (New England Biolabs, Hitchin, UK) restriction enzymes, according to the manufacturer’s instruction. The RFLP restriction patterns of the *gap* gene of staphylococci, after digestion with *Alu*I and *Hpy*CH4V enzymes, were published previously [43,47,48] and used for interpretation the results obtained for the investigated isolates. 

Control strains for the species identification of *Staphylococcus* included: *Staphylococcus xylosus* PCM 2114 (PCM, Polish Collection of Microorganisms), *S. equorum* PCM 2487, *S. arlettae* PCM 2528, *S. warneri* PCM 2107, *S. lentus* PCM 2441, *S. succinus* ATCC 700337 and *S. sciuri* PCM 2424.

### 2.7. Electrophoresis

The PCR and PCR-RFLP products were separated in 1.5% and 2% agarose gels (peqGOLD, Peqlab Biotechnologie GmbH, Erlangen, Germany), respectively, in 1X Tris-borate-EDTA (TBE) buffer (Bio-Rad, Hercules, USA), at 90 V for 45 min to 1 h, stained with 1% aqueous solution of ethidium bromide (Merck, Darmstadt, Germany) and analyzed using GeneTools software (Syngene, Cambridge, UK). 

### 2.8. Antimicrobial Resistance

Resistance to antimicrobials was determined using the disk diffusion method according to the guidelines of the European Committee on Antimicrobial Susceptibility Testing [49]. The following antimicrobial discs were used (Oxoid, UK): cefoxitin (30 µg), norfloxacin (10 µg), ciprofloxacin (5 µg), amikacin (30 µg), gentamicin (10 µg), tigecycline (15 µg), tetracycline (30 µg), linezolid (10 µg), sulfamethoxazole/trimethoprim (25 µg), rifampicine (5 µg) and chloramphenicol (30 µg). Macrolide–lincosamide–streptogramin B (MLS_B_) and macrolide–streptogramin B (MS_B_) resistance phenotypes were investigated by the double-disk diffusion test with erythromycin (15 µg) and clindamycin (2 µg) according to EUCAST recommendations [50].

## 3. Results

### 3.1. Isolation and Species Identification of Staphylococci

A total of 127 Gram-positive and catalase-positive cocci were isolated from 61 sheep belonging to Świniarka (33 individuals) and Wrzosówka (28 individuals) breeds (Table 1 and Table 2). In all isolates, the *gap* gene was detected, which proved the identification of the genus *Staphylococcus*. All staphylococci were coagulase-negative and none of them possessed a clumping factor. The ability to decompose mannitol was observed in 124 (97.6%) isolates and β-hemolysis was detected in 5 (3.9%) of 127 staphylococci.

Based on PCR-RFLP of the *gap* gene using the *Alu*I enzyme, five different restriction patterns specific to *S. xylosus*, *S. arlettae*, *S. warneri*, *S. lentus* and *S. sciuri*, and one non-specific pattern characteristic to *S. equorum* and *S. succinus* species were obtained (Table 1). However, the restriction analysis of the *gap* gene performed with the *Hpy*CH4V enzyme in the second stage of the study enabled to distinguish *S. equorum* and *S. succinus* species (Table 1). 

From 1 to 4 staphylococcal isolates were obtained from one sheep. In total, seven different species were identified among 127 isolates (Table 1). The most frequent was *S. xylosus* (43 isolates, 33.9%), followed by *S. equorum* (37 isolates, 29.1%), *S. arlettae* (19 isolates, 15%), *S. warneri* (12 isolates, 9.4%), *S. lentus* (10 isolates, 7.9%), *S. succinus* (5 isolates, 3.9%) and *S. sciuri* (1 isolate, 0.8%) (Table 1). The distribution of *Staphylococcus* species varied among breeds.

A greater number and species diversity of staphylococci were found among isolates obtained from Świniarka (73 isolates, 7 species) than Wrzosówka (54 isolates, 4 species) (Table 1 and Table 2). The species most frequently isolated from Świniarka was *S. equorum* (23/73, 31.5%), whereas *S. xylosus* prevailed among the isolates obtained from Wrzosówka (28/54, 51.9%) (Table 2). Isolates of *S. lentus*, *S. succinus* and *S. sciuri* were found only in swabs derived from Świniarka (Table 1 and Table 2).

### 3.2. Prevalence and Distribution of Superantigen (SAg) Genes

Among the 127 isolates, 98 (77.2%) were SAg genes-positive (Table 3 and Table 4). The percentage of SAg genes-positive staphylococci isolated from Świniarka and Wrzosówka was comparable and equaled 75.3% (55/73 isolates) and 79.6% (43/54 isolates), respectively (Table 4). The individual isolates harbored from 1 to 7 of the 21 analyzed genes encoding SAgs. Most of the strains harbored one (30 isolates, 23.6%) or two (25, 19.7%) SAg genes. In other strains four (13, 10.2%), five (12, 9.4%), three (11, 8.7%), six (4, 3.1%) and seven (3, 2.4%) SAg genes were detected. In contrast, in 29 isolates (22.8%) SAgs genes were not detected.

The occurrence and number of particular SAg genes differed between staphylococcal species (Table 3, Table 4 and Table 5). The highest number of different SAgs genes was recorded among *S. equorum* isolates (16 out of 21genes) (Table 3). The most prevalent SAg genes were: *ser* (51 isolates, 40.2%) among staphylococcal enterotoxin (SE) genes and *selq* (38 isolates, 29.9% isolates) among staphylococcal enterotoxin-like (SEl) proteins (Table 4 and Table 5). The genes encoding exfoliative toxins A (*eta*) and D (*etd*) were detected in one (0.8% isolates) and five isolates (3.9%), respectively (Table 4 and Table 5). In turn, *tst-1* gene encoding toxic shock syndrome toxin 1 was found in 17 isolates (13.4%) (Table 4 and Table 5). The *selj* and *selp* genes were not present in any of the isolates (Table 4 and Table 5). 

The most prevalent *ser* gene was found in 32 out of 55 (58.2%) isolates obtained from Świniarka and in 19 out of 43 (44.2%) staphylococci collected from Wrzosówka. However, the highest number of different SAg genes was recorded for isolates derived from Wrzosówka (18 different SAg genes) as compared to the isolates from Świniarka (15 different SAg genes) (Table 4). 

### 3.3. Antimicrobial Resistance 

Among all isolates, 77 (60.6%) exhibited antimicrobial resistance (Table 6 and Table 7). Drug resistance was species but not the source of isolation dependent. Species that showed the highest percentages of resistance belonged to *S. sciuri* (1/1 isolates, 100%), *S. lentus* (9/10 isolates, 90%), *S. warneri* (10/12 isolates, 83.3%), *S. xylosus* (33/43 isolates, 76.7%) and *S. arlettae* (14/19 isolates, 73.7%). Lower percentages of resistance were recorded for *S. succinus* (2/5 isolates, 40%) and *S. equorum* (8/37 isolates, 21.6%).

As regards resistance of all isolates to the 13 antimicrobials used in this study, the most frequent resistance was observed to clindamycin (58 isolates, 45.7%), erythromycin (37 isolates, 29.1%), and rifampicin (26 isolates, 20.5%). In turn, none of the isolates demonstrated resistance to cefoxitin, norfloxacin, ciprofloxacin, amikacin, tigecycline, trimethoprim/sulfamethoxazole and chloramphenicol (Table 6). Based on the results of the “D-zone” test, in 23 isolates (18.1%), belonging to three species (*S. xylosus*, *S, arlettae* and *S. warneri*), the mechanism of constitutive MLS_B_ resistance was detected. Moreover, the inducible MLS_B_ phenotype was demonstrated in six isolates (4.7%) belonging to three species (*S. xylosus*, *S. arlettae* and *S. lentus*) (Table 7). Additionally, in nine isolates (7.1%) belonging to two species (*S. xylosus* and *S. equorum*) resistance to erythromycin, indicating resistance to macrolide-streptogramin B antibiotics (MS_B_) was detected. Furthermore, in 35 isolates (27.6%) belonging to six species (*S. xylosus*, *S. equorum*, *S. arlettae*, *S. warneri, S. lentus and S. sciuri*) rare resistance to clindamycin was noted (L phenotype). Concerning the multiple resistances, 47 isolates (37%) belonging to *S. xylosus* (33 isolates) and *S. arlettae* (14 isolates) exhibited even six antimicrobial resistance phenotypes. In total, in 77 antimicrobial-resistant isolates, nine different phenotypes were identified (Table 7).

Evaluation of methicillin sensitivity observed in disk diffusion test was confirmed genetically. None of the isolates harbored the *mec*A gene.

## 4. Discussion

Staphylococci are common commensal microorganisms that colonize the skin and mucous membranes [25,51]. However, it should be emphasized that these bacteria, both *S. aureus* and CoNS, can also constitute the pathogens with particular importance in the etiology of various infections e.g., skin, wounds, ears, eyes, urinary tract, endocardium, joints, bloodstream, surgical site as well as mastitis and invasive device-related infections in both animals and humans [25,51,52,53,54]. In recent years, especially CoNS have been of great interest as pathogens [54]. However, the occurrence of CoNS in animals is still based mainly on mastitis cases [52,53,55,56]. On the other hand, it can also be noted that currently, the CoNS are taking the scope of research to gain ground against *S. aureus* in veterinary medicine [54]. It should be emphasized that the research on nasal colonization of staphylococci in animals, especially CoNS, and the clinical importance of this phenomenon are often underestimated [30]. Bearing in mind the importance of this issue, the current study focuses on the presence of staphylococci in healthy sheep and their toxicogenic potential and resistance to antimicrobial agents. 

The abundance and diversity of the microbiome are shaped by various factors. Recent studies indicate that the environment as well as the genetic and individual features play a role in the development of microbiota of ruminants. However, it is considered that relatively few studies have been conducted investigating the factors which could impact on the microbiota. Thus, studies focusing on host specific microbiotas are required [57,58]. Our results confirm a genetic determination rather than environmental influences on microbiome composition. This can be concluded because of the identification of only seven species of staphylococci among 127 staphylococci isolated from 61 sheep housed under the same conditions. On the other hand, the presence of 1 to 4 different staphylococci of various species from one animal may indicate individually determined conditions.

It should be noted that studies on the microbiota inhabiting the nasal cavity of healthy sheep are scarce. Queen et al. [59] gathered nasal and tonsillar samples from 14 free-ranging clinically healthy Rocky Mountain bighorn sheep and 10 domestic sheep. The authors identified 194 different bacterial isolates. *Staphylococcus* species (31 isolates) were the most numerous Gram-positive bacteria and had a higher incidence in samples from domestic (22 isolates) than from bighorn (9 isolates) animals. Among staphylococci isolated from nasal swabs, *S. aureus* and *S. simulans* were isolated only from domestic sheep, *S. cohnii* and *S. epidermidis* from bighorn sheep while *S. sciuri*, *S. warneri* and *S. xylosus* were isolated from both sheep. 

In turn, Jauro et al. [60] investigated 100 nasal swab samples collected from healthy and sick sheep. In their study, the rate of nasal carriage of *S. aureus* and MRSA in sheep was found to be 61% and 26%, respectively. The authors reported that 48 (73%) of the sick and 13 (38%) of the healthy sheep were positive for *S. aureus* and 24 (36%) of the sick sheep and 2 (6%) of the healthy sheep were found to harbor MRSA. Isolation of *S. aureus* from the nares of sheep has been also reported by other authors. In this context, Vautor at. al. [61] noticed the *S. aureus* nasal carriage in 29% of ewes of several dairy sheep breeds in farms producing cheeses manufactured from raw ewe’s milk. In turn, Gharsa et al. (2012) detected MRSA in 5 (3%) and MSSA in 68 (41.7%) nasal swabs obtained from 163 healthy sheep. Furthermore, Rahimi et al. [62] detected nasal carriage of *S. aureus* in 11 (14.1%) of 78 healthy sheep (one of these isolates harbored the *mec*A gene). 

Contrary to these reports, in the present study, no *S. aureus* or other coagulase-positive staphylococci (CPS) were identified. On the other hand, our results concerning the presence of *S. xylosus, S. warneri* and *S. sciuri* species in sheep nostrils along with *S. xylosus* predomination stay in line with those reported by Queen et al. [59]. Similarly, Rich [25], among staphylococci isolated from sheep, also reported the highest percentage of *S. xylosus,* however, according to these authors’ research, two other species namely *S. vitulinus* and *S. lentus* were also isolated in large numbers. In our work, *S. lentus* was also identified in 10 out of 33 Świniarka sheep. 

Abdel-Moein and Zaher [30] investigated the nasal carriage of CoNS among 152 different domestic and farm animals including 29 sheep (17 healthy and 12 with respiratory disease), and their public health implication. The authors identified CoNS in over 10% of sheep. Moreover, they also found, that none of the isolated staphylococci harbored drug resistance (*mec*A and *bla*Z) and/or virulence (*lukS/F-PV* and *tsst-1*) genes. Likewise, in our research, none of the isolated CoNS possessed the *mec*A gene, but in contrast to the above-mentioned report, in our study over 13% of isolates harbored the *tsst-1* gene. The current study has also shown, that out of 127 CoNS over 77% were SAg genes-positive and possessed from 1 to 7 of the 21 analyzed genes encoding SEs, SEls, ETA, ETD, and TSST-1. The highest number of different SAgs genes was recorded among *S. equorum* species and the most prevalent SAg gene was *ser* (over 40% of isolates harbored this gene). The possession of these virulence factors could be associated with increased pathogenicity of staphylococci [54], both *S. aureus* and CoNS [55,63,64]. Based on bibliographic sources, such a high percentage of potentially toxigenic staphylococcal isolates as reported in the current study is characteristic rather for *S. aureus* than for CoNS. This is consistent with the findings of Gharsa et al. [65] who confirmed that the nares of healthy sheep could be a reservoir of PVL-positive MRSA and of TSST-positive *S. aureus* isolates, with potential implications for public health. However, the findings of our study agreed with the reports of other researchers who demonstrated that CoNS isolated from different sources can harbor multiple SAgs genes [43,44,47,55,64]. 

Primitive breeds kept on organic farms get sick less frequently as compared to conventional farms or even more large-scale breeding [32]. Their treatment is based primarily on the use of natural substances of plant origin e.g., the essential oils (based on thyme, cinnamon, lemongrass, oregano, and rosemary) [66,67] and herbs (garlic, onion, dill, parsley, mint) which constitute an alternative to antibiotics and other conventional antimicrobial drugs [68]. All the animals included in our study were healthy and had never been treated with any antimicrobials or antiseptics. For this reason, it was assumed that the staphylococci isolated from such animals would be not only non-toxigenic but also sensitive to at least most antimicrobial agents. 

Contrary to our assumption, the study showed, that over 60% of CoNS were resistant to at least one antibiotic of therapeutic importance (gentamicin, erythromycin, clindamycin, tetracycline, rifampicin, or linezolid). Nevertheless, our findings are consistent with results obtained by Turchi et al. [69] and Holko et al. [70] who found that respectively over 53% and 63% of CoNS were resistant to at least one antimicrobial agent. Nevertheless, it should be emphasized that in both studies, CoNS were isolated from the milk of healthy sheep, not from their nostrils, and the animals were kept on conventional sheep farms.

In turn, Burriel [71] showed resistance among 75% of 83 CoNS isolated from milk of sheep with subclinical mastitis and from the teat skin of healthy ewes to a variety of 24 commonly used in human and veterinary medicine antimicrobial agents. Resistance was most common to trimethoprim, sulphonamide, co-trimoxazole, tetracycline, and chloramphenicol. The author also detects resistance to methicillin in four *S. epidermidis* and three *S. xylosus* isolates obtained from the milk of dairy ewes.

Interestingly, among the collected CoNS, we found that 35 isolates were resistant to clindamycin while being sensitive to erythromycin (L phenotype). The L phenotype resistance is reported more frequently in *Streptococcus* [72,73,74,75] than in *Staphylococcus*. However, recently, the detection of this unusual resistance phenotype has increased among staphylococcal strains of animal origin [76,77]. This result is particularly important because clindamycin is the most clinically important lincosamide, often used in veterinary medicine to treat infections caused by staphylococci (especially MRSA) [78,79,80]. Moreover, in the present study inducible (iMLS_B_) and constitutive (cMLS_B_) macrolide-lincosamide-streptogramin B resistance was detected in 29 (22.8%) isolates. Simultaneously, the inducible MLS_B_ and constative MLS_B_ resistant strains were the only multidrug-resistant (resistant to at least one agent in three or more antimicrobial categories) bacteria isolated in the present study. Additionally, nine isolates exhibited the MS_B_ phenotype. Currently, from the clinical point of view, macrolide-lincosamide-streptogramin B resistance is an increasing therapeutic complication [81,82]. The inducible MLS_B_ phenotype is detected both, in pathogenic and nonpathogenic *Staphylococcus* species isolated from humans and animals. It has been shown that CoNS inhabiting animals can display a wide range of antimicrobial resistance, and thus may potentially serve as a reservoir of resistance genes [51]. Therefore, the bacteria isolated in the current study could play a role as a reservoir for resistance genes [83]. 

The question remains, how did strains with the MLS_B_ resistance mechanism become a part of the physiological microbiota of sheep from an organic farm, never treated with any antimicrobials? Research done by Heß and Gallert [83] may provide a potential explanation. In their study, staphylococci isolated from sewage and river water (among them such CoNS species as *S. xylosus*, *S. sciuri*, and *S. lentus*) were tested for antibiotic resistance. The authors demonstrated that 13.7% *Staphylococcus* isolates were constitutively resistant to clindamycin and 16.5% revealed an inducible clindamycin resistance. Moreover, the percentage of isolates resistant to erythromycin and clindamycin was higher in the case of the bacteria isolated from the river than from the tertiary-treated sewage. The authors also reported that the concentration of erythromycin and anhydroerythromycin (degradation product of the erythromycin) in surface water was high enough to induce resistance to clindamycin in *Staphylococcus* strains (even after only 10 min of exposure). The antibiotic resistance mechanism is well studied in clinics, however, in aquatic environments, the resistance situation is rarely investigated. Nonetheless, it is well known that antibiotics and antibiotic-resistant bacteria are present in the natural environment, the especially aquatic environment which is polluted with antibiotics from human use [84,85]. Sheep included in our study are grazing in wetlands, near the Odra river. In Poland, the problem of antibiotics in wastewater is hardly monitored, however, there are reports on antibiotic concentrations in surface waters or bottom sediments, including the Odra river, proving the presence of macrolides and lincosamides [86,87]. Hence, the hypothesis may be posed, that physiological microbiota of sheep become resistant to clindamycin and erythromycin due to antibiotics present in surface waters. However, that hypothesis would require further studies. Another possible explanation is that antibiotic-resistant CoNS could be potentially acquired from a variety of other animals present in the sheep environment, such as chickens, goats, cattle, wild birds, and rodents [32,33]. Staphylococci may be transferred between different animals by direct contact, with excretions (sneezing, coughing, or licking), and via indirect transmission through contaminated environment or aerosols [33]. Regarding the other clinically important antimicrobials, all staphylococci isolated in the current study were sensitive to ciprofloxacin, amikacin, norfloxacin, tigecycline, chloramphenicol, and trimethoprim-sulfamethoxazole. Moreover, we did not find any methicillin-resistant isolates which is consistent with other authors’ studies, reporting a low percentage of methicillin-resistant isolates [56,88]. However, most of the reports included CoNS isolated from ovine milk of healthy sheep or animals with subclinical mastitis, and not from the nostrils of sheep. The observed susceptibility to methicillin among all isolates could be considered a positive result, considering the frequent occurrence of CoNS in dairy products which could serve as a carrier of methicillin-resistant strains from animals to consumers.

## 5. Conclusions

Our study has shown that toxigenic potential and resistance to antimicrobials is associated with bacteria colonizing the nasal cavity of healthy sheep from an organic farm. This finding indicates that staphylococci colonizing the nasal cavity of healthy sheep are the reservoir of genes encoding enterotoxins synthesis and antimicrobial resistance. Therefore, close contact with those animals or consuming dairy products made from their milk may potentially pose a threat to public health. 

What is important, *S. aureus* was not found among isolated staphylococci, which reveals the minor importance of the carriage of this species for this population of animals.

Interestingly, it was found that although most of the isolated CoNS species were found in both analyzed breeds of sheep, some of the isolated staphylococci showed species specificity related strictly to the breed.

## Figures and Tables

**Table 1:** Identification of staphylococci isolated from Świniarka and Wrzosówka breed.

PCR-RFLP Identification		Source of Isolation-Sheep Breed (No. of Isolates)		
*Alu*I	*Hpy*CH4V	Świniarka (33)	Wrzosówka (28)	Świniarka + Wrzosówka (61)
*Staphylococcus* Species		No. of Isolates (%)		
*S. xylosus*	*S. xylosus*	15 (34.9)	28 (65.1)	43 (33.9)
*S. equorum/S. succinus*	*S. equorum*	23 (62.2)	14 (37.8)	37 (29.1)
*S. arlettae*	*S. arlettae*	17 (89.5)	2 (10.5)	19 (15)
*S. warneri*	*S. warneri*	2 (16.7)	10 (83.3)	12 (9.4)
*S. lentus*	*S. lentus*	10 (100)	0 (0)	10 (7.9)
*S. equorum/S. succinus*	*S. succinus*	5 (100)	0 (0)	5 (3.9)
*S. sciuri*	*S. sciuri*	1 (100)	0 (0)	1 (0.8)
		**73 (57.5)**	**54 (42.5)**	**127 (100)**

**Table 2:** Species diversity vs. a source of isolation of staphylococci.

Sheep Breed	No. of Sheep (%)	*Staphylococcus* Species	No. of Isolates (%)	
**Świniarka**	33 (54.1)	*S. equorum*	23 (31.5)	73 (57.5)
		*S. arlettae*	17 (23.3)	
		*S. xylosus*	15 (20.5)	
		*S. lentus*	10 (13.7)	
		*S. succinus*	5 (6.8)	
		*S. warneri*	2 (2.7)	
		*S. sciuri*	1 (1.4)	
**Wrzosówka**	28 (45.9)	*S. xylosus*	28 (51.9)	54 (42.5)
		*S. equorum*	14 (25.9)	
		*S. warneri*	10 (18.5)	
		*S. arlettae*	2 (3.7)	
	**61 (100)**			**127 (100)**

**Table 3:** Distribution of superantigen (SAg) genes profile among *Staphylococcus* species.

Species	No. of SAg Genes-Positive Isolates (%)	SAg Genes (No. of Isolates)	No. of SAg Genes
*S. xylosus*	34/43 (79.1)	ser (15), seg (14), selq (13), selm (9), selo (9), sed (7), tst-1 (7), sei (6), sec (4), sea (3), seh (2), seln (2), see (1), sell (1), etd (1)	15
*S. equorum*	26/37 (70.3)	ser (12), seg (11), selm (11), selq (10), sei (7), selo (6), seln (4), sea (3), tst-1 (3), seb (1), sec (1), see (1), sell (1), selk (1), eta (1), etd (1)	16
*S. arlettae*	14/19 (73.7)	ser (9), selm (8), selq (5), sec (3), tst-1 (3), sea (2), seg (2), etd (2), sei (1), sed (1), sell (1), selo (1)	12
*S. warneri*	9/12 (75)	ser (6), selq (4), seg (2), selm (2), selo (2), tst-1 (2), sei (1), sell (1)	8
*S. lentus*	9/10 (90)	ser (7), selm (6), seg (5), selq (4), sei (3), sec (2), selo (2), tst-1 (2), sell (1), seln (1), selu (1), etd (1)	12
*S. succinus*	5/5 (100)	seg (2), ser (2), selk (1), selm (1), selo (1), selq (1)	6
*S. sciuri*	1/1 (100)	selq (1)	1
	**98/127 (77.2)**		

**Table 4:** Relationship between source of isolation and prevalence of superantigen (SAg) genes among *Staphylococcus* species.

Source of Isolation No. of Sheep (%)	Species (No. of Isolates)	No. of SAg Gene-Positive Isolates	*Ser*	*Selq*	*Selm*	*Seg*	*Selo*	*Sei*	*Tst-1*	*Sec*	*Sea*	*Sed*	*Seln*	*Sell*	*Etd*	*See*	*Seh*	*Selk*	*Seb*	*Selu*	*Eta*	*Selj*	*Selp*
**Świniarka 33 (54.1)**	*S. equorum* (23)	16	7	4	8	6	5	5	3	1	2		3	1	1								
	*S. arlettae* (17)	12	8	5	7	2			2	2	2	1		1	1								
	*S.* xylosus (15)	11	7	4	1	4	2		3	2		2	1										
	*S.* lentus (10)	9	7	4	6	5	2	3	2	2			1	1	1					1			
	*S.* succinus (5)	5	2	1	1	2	1											1					
	*S.* warneri (2)	1	1	1		1																	
	*S.* sciuri (1)	1		1																			
	**73**	**55**	**32**	**20**	**23**	**20**	**10**	**8**	**10**	**7**	**4**	**3**	**5**	**3**	**3**			**1**		**1**			
**Wrzosówka 28 (45.9)**	*S.* xylosus (28)	23	8	9	8	10	7	6	4	2	3	5	1	1	1	1	2						
	*S.* equorum (14)	10	5	6	3	5	1	2			1		1			1		1	1		1		
	*S.* warneri (10)	8	5	3	2	1	2	1	2					1									
	*S. arlettae* (2)	2	1		1		1	1	1	1					1								
	**54**	**43**	**19**	**18**	**14**	**16**	**11**	**10**	**7**	**3**	**4**	**5**	**2**	**2**	**2**	**2**	**2**	**1**	**1**		**1**		
**61 (100)**	**127 (100)**	**98 (77.2)**	**51**	**38**	**37**	**36**	**21**	**18**	**17**	**10**	**8**	**8**	**7**	**5**	**5**	**2**	**2**	**2**	**1**	**1**	**1**	**0**	**0**

**Table 5:** Prevalence of superantigen (SAg) genes among *Staphylococcus* species.

Species (No. of Isolates)	SAg Genes																				
	*Ser*	*Selq*	*Selm*	*Seg*	*Selo*	*Sei*	*Tst-1*	*Sec*	*Sea*	*Sed*	*Seln*	*Sell*	*Etd*	*See*	*Seh*	*Selk*	*Seb*	*Selu*	*Eta*	*Selj*	*Selp*
	No. of SAg Gene-Positive Isolates																				
*S.* xylosus (43)	15	13	9	14	9	6	7	4	3	7	2	1	1	1	2	0	0	0	0	0	0
*S. equorum* (37)	12	10	11	11	6	7	3	1	3	0	4	1	1	1	0	1	1	0	1	0	0
*S. arlettae* (19)	9	5	8	2	1	1	3	3	2	1	0	1	2	0	0	0	0	0	0	0	0
*S. warneri* (12)	6	4	2	2	2	1	2	0	0	0	0	1	0	0	0	0	0	0	0	0	0
*S. lentus* (10)	7	4	6	5	2	3	2	2	0	0	1	1	1	0	0	0	0	1	0	0	0
*S. succinus* (5)	2	1	1	2	1	0	0	0	0	0	0	0	0	0	0	1	0	0	0	0	0
*S. sciuri* (1)	0	1	0	0	0	0	0	0	0	0	0	0	0	0	0	0	0	0	0	0	0
	**51**	**38**	**37**	**36**	**21**	**18**	**17**	**10**	**8**	**8**	**7**	**5**	**5**	**2**	**2**	**2**	**1**	**1**	**1**	**0**	**0**

**Table 6:** Relationship between a source of isolation and antimicrobial resistance of staphylococci.

Source of Isolation (No. of Sheep)	Species (No. of Isolates)	No. of Resistant Isolates (%)	Antimicrobial												
			No. of Resistant Isolates												
			FOX	E	DA	NOR	CIP	AK	CN	TGC	TE	LZD	SXT	RD	C
**Świniarka breed** **(33)**	*S. equorum* (23)	4/23 (17.4)	0	4	0	0	0	0	0	0	0	0	0	0	0
	*S. arlettae* (17)	13/17 (76.5)	0	5	10	0	0	0	1	0	1	0	0	2	0
	*S. xylosus* (15)	11/15 (73.3)	0	5	9	0	0	0	0	0	0	0	0	4	0
	*S. lentus* (10)	9/10 (90)	0	1	8	0	0	0	0	0	0	2	0	0	0
	*S. succinus* (5)	2/5 (40)	0	0	0	0	0	0	0	0	0	0	0	2	0
	*S. warneri* (2)	2/2 (100)	0	2	2	0	0	0	0	0	0	0	0	2	0
	*S. sciuri* (1)	1/1 (100)	0	0	1	0	0	0	0	0	0	0	0	0	0
	**Total no. 73**	**42/73 (57.5)**	**0**	**17**	**30**	**0**	**0**	**0**	**1**	**0**	**1**	**2**	**0**	**10**	**0**
**Wrzosówka breed (28)**	*S. xylosus* (28)	22/28 (78.6)	0	12	19	0	0	0	0	0	0	0	0	9	0
	*S. equorum* (14)	4/14 (28.6)	0	4	0	0	0	0	0	0	0	0	0	0	0
	*S. warneri* (10)	8/10 (80)	0	4	8	0	0	0	0	0	0	0	0	6	0
	*S. arlettae* (2)	1/2 (50)	0	0	1	0	0	0	0	0	0	0	0	0	0
	**Total no. 54**	**35/54 (64.8)**	**0**	**20**	**28**	**0**	**0**	**0**	**0**	**0**	**0**	**0**	**0**	**15**	**0**
	**127**	**77/127 (60.6)**	**0**	**37**	**58**	**0**	**0**	**0**	**1**	**0**	**1**	**2**	**0**	**26**	**0**

FOX—cefoxitin, E—erythromycin, DA—clindamycin, NOR—norfloxacin, CIP—ciprofloxacin, AK—amikacin, CN—gentamicin, TGC—tigecycline, TE—tetracycline, LZD—linezolid, SXT—trimetoprim/sulfamethoxazole, RD—rifampicin, C—chloramphenicol.

**Table 7:** Antibiotic resistance of staphylococci.

Species	No. of Isolates	Resistant Isolates (%)	Resistance Phenotypes (No. of Isolates)	Resistance Mechanisms (No. of Isolates)
*S. xylosus*	43	33/43 (76.7)	DA-RD (3), E (3), DA (11), E-DA (6), RD (2), E-DA-RD (8)	cMLS_B_ (14), iMLSB (3), MSB (1), L phenotype (14)
*S. equorum*	37	8/37 (21.6)	E (8)	MSB (8)
*S. arlettae*	19	14/19 (73.7)	CN-DA (1), E (2), TE (1), E-DA (1), DA (7), E-DA-RD (2)	cMLSB (3), iMLSB (2), L phenotype (8)
*S. warneri*	12	10/12 (83.3)	E-DA-RD (6), DA-RD (2), DA (2)	cMLSB (6), L phenotype (4)
*S. lentus*	10	9/10 (90)	DA (6), DA-LZD (2), E (1)	iMLSB (1), L phenotype (8)
*S. succinus*	5	2/5 (40)	RD (2)	-
*S. sciuri*	1	1/1 (100)	DA (1)	L phenotype (1)
**Total no.**	**127**	**77 (60.6)**		

CN—gentamicin, DA—clindamycin, E—erythromycin, LZD—linezolid, RD—rifampicin, TE—tetracycline.

## Data Availability

All obtained data from this study were included in the manuscript.

## References

[1] Dagnew Y., Urge M., Tadesse Y., Gizaw S. (2017). Sheep Production and Breeding Systems in North Western Lowlands of Amhara Region, Ethiopia: Implication for Conservation and Improvement of Gumz Sheep Breed. Open J. Anim. Sci..

[2] Marsoner T., Egarter Vigl L., Manck F., Jaritz G., Tappeiner U., Tasser E. (2018). Indigenous Livestock Breeds as Indicators for Cultural Ecosystem Services: A Spatial Analysis within the Alpine Space. Ecol. Indic..

[3] Molotsi A.H., Dube B., Cloete S.W.P. (2019). The Current Status of Indigenous Ovine Genetic Resources in Southern Africa and Future Sustainable Utilisation to Improve Livelihoods. Diversity.

[4] Polak G., Krupiński J., Martyniuk E., Calik J., Kawęcka A., Krawczyk J., Majewska A., Sikora J., Sosin-Bzducha E., Szyndler-Nędza M. (2021). The Risk Status of Polish Local Breeds under Conservation Programmes—New Approach. Ann. Anim. Sci..

[5] Sobala M. (2014). Pasture Landscapes in Poland and Europe—Selected Types, Examples and Conservation Methods. Disserations Cult. Landsc. Commision.

[6] Gruszecki T.M., Warda M., Kulik M., Junkuszew A., Patkowski K., Bojar W., Tomczuk K., Greguła-Kania M., Dudko E., Bielińska E.J. (2017). Sheep Grazing to Protect the Diversity of Plant Communities in Valuable Natural Habitats. Wiadomości Zootech..

[7] Piestrzynska-Kajtoch A., Smołucha G., Oczkowicz M., Kycko A., Polak M.P., Kozaczyński W., Kozubska-Sobocińska A., Żmudziński J.F., Rejduch B. (2017). The Reference Gene Selection to Study *PRNP* Gene Expression in Sheep. Folia Biol..

[8] Chodkiewicz A. (2020). Advantages and Disadvantages of Polish Primitive Horse Grazing on Valuable Nature Areas—A Review. Glob. Ecol. Conserv..

[9] Junkuszew A., Milerski M., Bojar W., Szczepaniak K., le Scouarnec J., Tomczuk K., Dudko P., Studzińska M.B., Demkowska-Kutrzepa M., Bracik K. (2015). Effect of Various Antiparasitic Treatments on Lamb Growth and Mortality. Small Rumin. Res..

[10] Ross L.C., Austrheim G., Asheim L.-J., Bjarnason G., Feilberg J., Fosaa A.M., Hester A.J., Holand Ø., Jónsdóttir I.S., Mortensen L.E. (2016). Sheep Grazing in the North Atlantic Region: A Long-Term Perspective on Environmental Sustainability. Ambio.

[11] Kawęcka A., Pasternak M., Miksza-Cybulska A., Puchała M. (2022). Native Sheep Breeds in Poland—Importance and Outcomes of Genetic Resources Protection Programmes. Animals.

[12] IZPIB (2022). Genetic Resource Protection Programs Livestock. Http://Www.Bioroznorodnosc.Izoo.Krakow.Pl/.

[13] Kawęcka A., Knapik J. (2015). Użytkowość Mięsna Jagniąt Rodzimych Ras Owiec - Świniarki i Wrzosówki. Rocz. Nauk. Zootech..

[14] Klepacki B., Rokicki T. (2019). The Situation of Sheep Farms in Podlasie Region with Special Regard to Conservation Breeds of Sheep. Small Agric. Hold..

[15] Gurgul A., Jasielczuk I., Miksza-Cybulska A., Kawęcka A., Szmatoła T., Krupiński J. (2021). Evaluation of Genetic Differentiation and Genome-Wide Selection Signatures in Polish Local Sheep Breeds. Livest. Sci..

[16] Kawęcka A., Radkowska I., Kawęcka A. (2018). Meat Quality and Slaughter Traits of Native Świniarka Lambs Depending on a Housing System. J. Elem..

[17] Longo M.L., Vargas Junior F.M., Cansian K., Souza M.R., Burim P.C., Silva A.L.A., Costa C.M., Seno L.O. (2018). Environmental Factors That Influence Milk Production of Pantaneiro Ewes and the Weight Gain of Their Lambs during the Pre-Weaning Period. Trop. Anim. Health Prod..

[18] Kiec W. (2000). The Productivity of Polish Wrzosówka Sheep in Conditions of Preservation. Anim./Recur. Genét. Anim..

[19] Radko A., Rychlik T., Słota E. (2006). Genetic Characterization of the Wrzosówka Sheep Breed on the Basis of 14 Microsatellite DNA Markers. Med. Wet..

[20] Kawęcka A., Sosin-Bzducha E., Sikora J. (2016). Evaluation of Carcass and Meat Quality in Native Wrzosówka Lambs Fed Linseed-Supplemented Diet. Zywnosc Nauka Technol. Jakosc/Food Sci. Technol. Qual..

[21] PZO (2022). Polish Union of Sheep-Farmers. Http://Pzow.Pl/.

[22] Di Trana A., Sepe L., di Gregorio P., di Napoli M.A., Giorgio D., Caputo A.R., Claps S. (2015). The Role of Local Sheep and Goat Breeds and Their Products as a Tool for Sustainability and Safeguard of the Mediterranean Environment. The Sustainability of Agro-Food and Natural Resource Systems in the Mediterranean Basin.

[23] Papachristoforou C., Koumas A., Hadjipavlou G. (2013). Adding Value to Local Breeds with Particular Reference to Sheep and Goats. Anim. Genet. Resour./Resour. Génét. Anim./Recur. Genét. Anim..

[24] Kayili E., Sanlibaba P. (2020). Prevalence, Characterization and Antibiotic Resistance of *Staphylococcus aureus* Isolated from Traditional Cheeses in Turkey. Int. J. Food Prop..

[25] Rich M. (2005). Staphylococci in Animals: Prevalence, Identification and Antimicrobial Susceptibility, with an Emphasis on Methicillin-Resistant *Staphylococcus aureus*. Br. J. Biomed. Sci..

[26] Chajęcka-Wierzchowska W., Zadernowska A., Nalepa B., Sierpińska M., Łaniewska-Trokenheim Ł. (2014). Retail Ready-to-Eat Food as a Potential Vehicle for *Staphylococcus* spp. Harboring Antibiotic Resistance Genes. J. Food Prot..

[27] Şanlıbaba P. (2022). Prevalence, Antibiotic Resistance, and Enterotoxin Production of *Staphylococcus aureus* Isolated from Retail Raw Beef, Sheep, and Lamb Meat in Turkey. Int. J. Food Microbiol..

[28] Wang X., Wang X., Wang Y., Guo G., Usman T., Hao D., Tang X., Zhang Y., Yu Y. (2014). Antimicrobial Resistance and Toxin Gene Profiles of *Staphylococcus aureus* Strains from Holstein Milk. Lett. Appl. Microbiol..

[29] Bagcigil F.A., Moodley A., Baptiste K.E., Jensen V.F., Guardabassi L. (2007). Occurrence, Species Distribution, Antimicrobial Resistance and Clonality of Methicillin- and Erythromycin-Resistant Staphylococci in the Nasal Cavity of Domestic Animals. Vet. Microbiol..

[30] Abdel-Moein K.A., Zaher H.M. (2020). The Nasal Carriage of Coagulase-Negative Staphylococci Among Animals and Its Public Health Implication. Vector-Borne Zoonotic Dis..

[31] Abed A., Hamed N., Abd El Halim S. (2022). Coagulase Negative Staphylococci Causing Subclinical Mastitis in Sheep: Prevalence, Phenotypic and Genotypic Characterization. J. Vet. Med. Res..

[32] Bhargava K., Zhang Y. (2012). Multidrug-Resistant Coagulase-Negative Staphylococci in Food Animals. J. Appl. Microbiol..

[33] Schwarz S., Feßler A.T., Loncaric I., Wu C., Kadlec K., Wang Y., Shen J. (2018). Antimicrobial Resistance among Staphylococci of Animal Origin. Microbiol. Spectr..

[34] Taponen S., Pyörälä S. (2009). Coagulase-Negative Staphylococci as Cause of Bovine Mastitis—Not so Different from *Staphylococcus aureus*?. Vet. Microbiol..

[35] Abied A., Bagadi A., Bordbar F., Pu Y., Augustino S.M.A., Xue X., Xing F., Gebreselassie G., Han J.-L., Mwacharo J.M. (2020). Genomic Diversity, Population Structure, and Signature of Selection in Five Chinese Native Sheep Breeds Adapted to Extreme Environments. Genes.

[36] Gavojdian D., Padeanu I., Sauer M., Dragomir N., Ilisiu E., Kusza S., Rahmann G. (2016). Effects of Using Indigenous Heritage Sheep Breeds in Organic and Low-Input Production Systems on Production Efficiency and Animal Welfare in Romania. Landbauforsch. Appl. Agric. For. Res..

[37] Caroprese M., Ciliberti M.G., Marino R., Napolitano F., Braghieri A., Sevi A., Albenzio M. (2020). Effect of Information on Geographical Origin, Duration of Transport and Welfare Condition on Consumer’s Acceptance of Lamb Meat. Sci. Rep..

[38] Yugueros J., Temprano A., Berzal B., Sánchez M., Hernanz C., Luengo J.M., Naharro G. (2000). Glyceraldehyde-3-Phosphate Dehydrogenase-Encoding Gene as a Useful Taxonomic Tool for *Staphylococcus* spp.. J. Clin. Microbiol..

[39] Murakami K., Minamide W., Wada K., Nakamura E., Teraoka H., Watanabe S. (1991). Identification of Methicillin-Resistant Strains of Staphylococci by Polymerase Chain Reaction. J. Clin. Microbiol..

[40] Zhang S., Iandolo J.J., Stewart G.C. (1998). The Enterotoxin D Plasmid of *Staphylococcus aureus* Encodes a Second Enterotoxin Determinant (*Sej*). FEMS Microbiol. Lett..

[41] Jarraud S., Mougel C., Thioulouse J., Lina G., Meugnier H., Forey F., Nesme X., Etienne J., Vandenesch F. (2002). Relationships between *Staphylococcus aureus* Genetic Background, Virulence Factors, *Agr* Groups (Alleles), and Human Disease. Infect. Immun..

[42] Holtfreter S., Grumann D., Schmudde M., Nguyen H.T.T., Eichler P., Strommenger B., Kopron K., Kolata J., Giedrys-Kalemba S., Steinmetz I. (2007). Clonal Distribution of Superantigen Genes in Clinical *Staphylococcus aureus* Isolates. J. Clin. Microbiol..

[43] Fijałkowski K., Peitler D., Karakulska J. (2016). Staphylococci Isolated from Ready-to-Eat Meat—Identification, Antibiotic Resistance and Toxin Gene Profile. Int. J. Food Microbiol..

[44] Fijałkowski K., Struk M., Karakulska J., Paszkowska A., Giedrys-Kalemba S., Masiuk H., Czernomysy-Furowicz D., Nawrotek P. (2014). Comparative Analysis of Superantigen Genes in *Staphylococcus xylosus* and *Staphylococcus aureus* Isolates Collected from a Single Mammary Quarter of Cows with Mastitis. J. Microbiol..

[45] Wu D., Li X., Yang Y., Zheng Y., Wang C., Deng L., Liu L., Li C., Shang Y., Zhao C. (2011). Superantigen Gene Profiles and Presence of Exfoliative Toxin Genes in Community-Acquired Meticillin-Resistant *Staphylococcus aureus* Isolated from Chinese Children. J. Med. Microbiol..

[46] Kuroda M., Ohta T., Uchiyama I., Baba T., Yuzawa H., Kobayashi I., Cui L., Oguchi A., Aoki K., Nagai Y. (2001). Whole Genome Sequencing of Meticillin-Resistant *Staphylococcus aureus*. Lancet.

[47] Karakulska J., Fijałkowski K., Nawrotek P., Pobucewicz A., Poszumski F., Czernomysy-Furowicz D. (2012). Identification and Methicillin Resistance of Coagulase-Negative Staphylococci Isolated from Nasal Cavity of Healthy Horses. J. Microbiol..

[48] Karakulska J., Fijałkowski K. (2014). In Silico Identification of 44 Species and Subspecies of Staphylococci by Restriction Analysis of the Gap Gene Polymorphism Using HpyCH4V Enzyme. Afr. J. Microbiol. Res..

[49] (2021). EUCAST Disk Diffusion Method for Antimicrobial Susceptibility Testing-Version 9.0. Https://Www.Eucast.Org/Ast_of_bacteria/Disk_diffusion_methodology/.

[50] The European Committee on Antimicrobial Susceptibility Testing (2021). Breakpoint Tables for Interpretation of MICs and Zone Diameters. Http://Www.Eucast.Org.

[51] Michels R., Last K., Becker S.L., Papan C. (2021). Update on Coagulase-Negative Staphylococci—What the Clinician Should Know. Microorganisms.

[52] Vasileiou N.G.C., Chatzopoulos D.C., Sarrou S., Fragkou I.A., Katsafadou A.I., Mavrogianni V.S., Petinaki E., Fthenakis G.C. (2019). Role of Staphylococci in Mastitis in Sheep. J. Dairy Res..

[53] Achek R., El-Adawy H., Hotzel H., Tomaso H., Ehricht R., Hamdi T.M., Azzi O., Monecke S. (2020). Short Communication: Diversity of Staphylococci Isolated from Sheep Mastitis in Northern Algeria. J. Dairy Sci..

[54] Asante J., Amoako D.G., Abia A.L.K., Somboro A.M., Govinden U., Bester L.A., Essack S.Y. (2020). Review of Clinically and Epidemiologically Relevant Coagulase-Negative Staphylococci in Africa. Microb. Drug Resist..

[55] Park J.Y., Fox L.K., Seo K.S., McGuire M.A., Park Y.H., Rurangirwa F.R., Sischo W.M., Bohach G.A. (2011). Detection of Classical and Newly Described Staphylococcal Superantigen Genes in Coagulase-Negative Staphylococci Isolated from Bovine Intramammary Infections. Vet. Microbiol..

[56] Martins K.B., Faccioli P.Y., Bonesso M.F., Fernandes S., Oliveira A.A., Dantas A., Zafalon L.F., Cunha M.d.L.R.S. (2017). Characteristics of Resistance and Virulence Factors in Different Species of Coagulase-Negative Staphylococci Isolated from Milk of Healthy Sheep and Animals with Subclinical Mastitis. J. Dairy Sci..

[57] Mamun M.A.A., Sandeman M., Rayment P., Brook-Carter P., Scholes E., Kasinadhuni N., Piedrafita D., Greenhill A.R. (2020). The Composition and Stability of the Faecal Microbiota of Merino Sheep. J. Appl. Microbiol..

[58] Cholewińska P., Czyż K., Nowakowski P., Wyrostek A. (2020). The Microbiome of the Digestive System of Ruminants—A Review. Anim. Health Res. Rev..

[59] Queen C., Ward A.C., Hunter D.L. (1994). Bacteria Isolated from Nasal and Tonsillar Samples of Clinically Healthy Rocky Mountain Bighorn and Domestic Sheep. J. Wildl. Dis..

[60] Jauro S., Hamman M.M., Malgwi K.D., Musa J.A., Ngoshe Y.B., Gulani I.A., Kwoji I.D., Iliya I., Abubakar M.B., Fasina F.O. (2022). Antimicrobial Resistance Pattern of Methicillin-Resistant *Staphylococcus aureus* Isolated from Sheep and Humans in Veterinary Hospital Maiduguri, Nigeria. Vet. World.

[61] Vautor E., Abadie G., Guibert J.-M., Chevalier N., Pépin M. (2005). Nasal Carriage of *Staphylococcus aureus* in Dairy Sheep. Vet. Microbiol..

[62] Rahimi H., Dastmalchi Saei H., Ahmadi M. (2015). Nasal Carriage of *Staphylococcus aureus*: Frequency and Antibiotic Resistance in Healthy Ruminants. Jundishapur J. Microbiol..

[63] Vasconcelos N.G., da Cunha M.d.L.R.S. (2010). Staphylococcal Enterotoxins: Molecular Aspects and Detection Methods. J. Public Health Epidemiol..

[64] Vasconcelos N.G., Pereira V.C., Araújo Júnior J.P., da Cunha M.d.L.R.S. (2011). Molecular Detection of Enterotoxins E, G, H and I in *Staphylococcus aureus* and Coagulase-Negative Staphylococci Isolated from Clinical Samples of Newborns in Brazil. J. Appl. Microbiol..

[65] Gharsa H., ben Slama K., Lozano C., Gómez-Sanz E., Klibi N., ben Sallem R., Gómez P., Zarazaga M., Boudabous A., Torres C. (2012). Prevalence, Antibiotic Resistance, Virulence Traits and Genetic Lineages of *Staphylococcus aureus* in Healthy Sheep in Tunisia. Vet. Microbiol..

[66] Ribeiro A.D.B., Ferraz Junior M.V.C., Polizel D.M., Miszura A.A., Gobato L.G.M., Barroso J.P.R., Susin I., Pires A.V. (2019). Thyme Essential Oil for Sheep: Effect on Rumen Fermentation, Nutrient Digestibility, Nitrogen Metabolism, and Growth. Arq. Bras. Med. Vet. Zootec..

[67] Ebani V.V., Mancianti F. (2020). Use of Essential Oils in Veterinary Medicine to Combat Bacterial and Fungal Infections. Vet. Sci..

[68] Zeineldin M., Abdelmegeid M., Barakat R., Ghanem M. (2018). A Review: Herbal Medicine as an Effective Therapeutic Approach for Treating Digestive Disorders in Small Ruminants. Alex. J. Vet. Sci..

[69] Turchi B., Bertelloni F., Marzoli F., Cerri D., Tola S., Azara E., Longheu C.M., Tassi R., Schiavo M., Cilia G. (2020). Coagulase Negative Staphylococci from Ovine Milk: Genotypic and Phenotypic Characterization of Susceptibility to Antibiotics, Disinfectants and Biofilm Production. Small Rumin. Res..

[70] Holko I., Tančin V., Tvarožková K., Supuka P., Supuková A., Lucia M. (2019). Occurence and Antimicrobial Resistance of Common Udder Pathogens Isolated from Sheep Milk in Slovakia. Potravin. Slovak J. Food Sci..

[71] Burriel A.R. (1997). Resistance of Coagulase-Negative Staphylococci Isolated from Sheep to Various Antimicrobial Agents. Res. Vet. Sci..

[72] De Azavedo J.C.S., McGavin M., Duncan C., Low D.E., McGeer A. (2001). Prevalence and Mechanisms of Macrolide Resistance in Invasive and Noninvasive Group B Streptococcus Isolates from Ontario, Canada. Antimicrob. Agents Chemother..

[73] Arana D.M., Rojo-Bezares B., Torres C., Alós J.I. (2014). First Clinical Isolate in Europe of Clindamycin-Resistant Group B Streptococcus Mediated by the Lnu(B) Gene. Rev. Esp. Quimioter.

[74] Hawkins P.A., Law C.S., Metcalf B.J., Chochua S., Jackson D.M., Westblade L.F., Jerris R., Beall B.W., McGee L. (2017). Cross-Resistance to Lincosamides, Streptogramins A and Pleuromutilins in Streptococcus Agalactiae Isolates from the USA. J. Antimicrob. Chemother..

[75] Zhou K., Zhu D., Tao Y., Xie L., Han L., Zhang Y., Sun J. (2019). New Genetic Context of Lnu(B) Composed of Two Multi-Resistance Gene Clusters in Clinical Streptococcus Agalactiae ST-19 Strains. Antimicrob. Resist. Infect. Control..

[76] Hauschild T., Fessler A.T., Kadlec K., Billerbeck C., Schwarz S. (2012). Detection of the Novel Vga(E) Gene in Methicillin-Resistant *Staphylococcus aureus* CC398 Isolates from Cattle and Poultry. J. Antimicrob. Chemother..

[77] Lozano C., Aspiroz C., Saenz Y., Ruiz-Garcia M., Royo-Garcia G., Gomez-Sanz E., Ruiz-Larrea F., Zarazaga M., Torres C. (2012). Genetic Environment and Location of the Lnu(A) and Lnu(B) Genes in Methicillin-Resistant *Staphylococcus aureus* and Other Staphylococci of Animal and Human Origin. J. Antimicrob. Chemother..

[78] Rich M., Deighton L., Roberts L. (2005). Clindamycin-Resistance in Methicillin-Resistant Isolated from Animals. Vet. Microbiol..

[79] Rezanka T., Spizek J., Sigler K. (2007). Medicinal Use of Lincosamides and Microbial Resistance to Them. Agents Med. Chem..

[80] Johnson M.D., Decker C.F. (2008). Antimicrobial Agents in Treatment of MRSA Infections. Dis. A-Mon..

[81] Deotale V., Mendiratta D., Raut U., Narang P. (2010). Inducible Clindamycin Resistance in *Staphylococcus aureus* Isolated from Clinical Samples. Indian J. Med. Microbiol..

[82] Miklasińska-Majdanik M. (2021). Mechanisms of Resistance to Macrolide Antibiotics among *Staphylococcus aureus*. Antibiotics.

[83] Heß S., Gallert C. (2014). Demonstration of Staphylococci with Inducible Macrolide-Lincosamide-Streptogramin B (MLS _B_) Resistance in Sewage and River Water and of the Capacity of Anhydroerythromycin to Induce MLS _B_. FEMS Microbiol. Ecol..

[84] Kummerer K. (2004). Resistance in the Environment. J. Antimicrob. Chemother..

[85] Larsson D.G.J., Flach C.-F. (2022). Antibiotic Resistance in the Environment. Nat. Rev. Microbiol..

[86] Szymańska U., Wiergowski M., Sołtyszewski I., Kuzemko J., Wiergowska G., Woźniak M.K. (2019). Presence of Antibiotics in the Aquatic Environment in Europe and Their Analytical Monitoring: Recent Trends and Perspectives. Microchem. J..

[87] Kucharski D., Nałęcz-Jawecki G., Drzewicz P., Skowronek A., Mianowicz K., Strzelecka A., Giebułtowicz J. (2022). The Assessment of Environmental Risk Related to the Occurrence of Pharmaceuticals in Bottom Sediments of the Odra River Estuary (SW Baltic Sea). Sci. Total Environ..

[88] Onni T., Sanna G., Larsen J., Tola S. (2011). Antimicrobial Susceptibilities and Population Structure of *Staphylococcus epidermidis* Associated with Ovine Mastitis. Vet. Microbiol..

